# Phenotypic effect of growth media on *Arabidopsis thaliana* root hair growth

**DOI:** 10.1080/15592324.2022.2104002

**Published:** 2022-08-24

**Authors:** Naomi Claeijs, Kris Vissenberg

**Affiliations:** a Integrated Molecular Plant Physiology Research (IMPRES); Biology Department, University of Antwerp, Antwerp, Belgium; b Plant Biochemistry & Biotechnology Lab, Department of Agriculture, Hellenic Mediterranean University, Heraklion, Greece

**Keywords:** *Arabidopsis thaliana*, gelling agent, growth media, *in vitro* growth, phenotype, root hair development

## Abstract

Over the years, many different growth media have been used to grow *Arabidopsis thaliana in vitro* in petri dishes. For these media the nutrient composition may vary, sugars may or may not be added, the medium may or may not be buffered and there is a choice between different gelling agents. The magnitude of possible combinations of these variables obstructs easy comparison of seedling phenotypes grown on the different media. This is especially obvious when it concerns the study of root hairs that are extremely sensitive to changes in their environment. To demonstrate this effect, we have grown *Arabidopsis thaliana* wild-type seeds on 18 different combinations of growth media and quantified root hair development. Comparison of root hair length and the respective root hair profiles identified the media that result in the formation of the longest root hairs. On these favored media they elongate through tip growth at a constant growth rate until they reach their final length (around 0.6 mm) at a distance of ±4 mm from the root tip.

## Introduction

Since the sequencing of the Arabidopsis genome,^1^ many efforts have been made to discover gene functions and to elucidate their role in development and growth responses. Since *in vivo* the root system develops in the soil, non-intrusive phenotyping poses severe practical challenges. Therefore, *in vitro* systems are frequently exploited to demonstrate root development and architectural plasticity.^2^ The use of semi-transparent media allows easier experimental manipulation and imaging of roots, and does this at a high spatio-temporal resolution, which is frequently requested.^3^ Plant cells have evolved complex regulatory networks in order to adapt to environmental changes and to respond to morphological or developmental stimuli. Root hairs, tubular extensions of specific epidermal cells, are single-cell model systems for the study of such regulatory networks. Over the years, many mutants have been phenotyped and characterized, and specific roles were attributed to certain genes.^4^ Strikingly, direct comparison of root hair phenotypes between studies is very difficult as there seems no similarity regarding the *in vitro* growth medium used. This addendum offers insights into the effect of different growth media on root hair development. In total, media of 18 different compositions were made and used to phenotype wild-type (Columbia-0, United States) root hairs.

## Effect of different growth media on root hair growth

**Figure 1.**
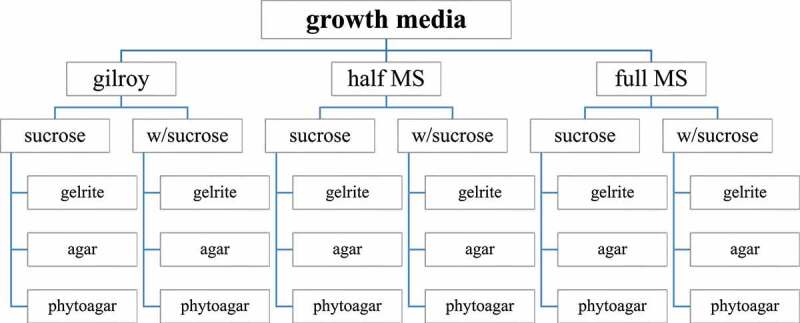
Graphical representation of the media that have been tested (18 different combinations: type of media, with or without (w/) sucrose, choice of gelling agent) .

As mentioned earlier, Arabidopsis has been grown over the years on multiple growth media. To demonstrate the effect of the choice of growth media on root hair growth, we have tested the effect of 18 different combinations on root hair growth (Figure 1). In this experiment, wild-type seeds (Columbia-0) were sown on 18 different petri round dishes. A distinction was made between the type of media, the presence of sucrose, and the type of gelling agent. After stratification for 3 days at 4°C, the petri dishes were placed vertically in a growth chamber at 21°C with a standard of 16/8 h light/dark photoperiod (*in vitro* conditions, direct illumination of shoot and root).^5^

After 6 days in the growth chamber (16/8 light/dark photoperiod), seedlings were photographed in the afternoon using a Nikon AZ-100 Multizoom microscope equipped with a Nikon DS-Ri1 digital camera. For each growth medium, the roots of the seedlings were photographed using a zoom magnification of 2x with the microscope’s 1x objective. Then the length of the root hairs was measured using ImageJ. Statistics were performed using the Rstudio statistics platform. In this manuscript, statistical inferences are not implemented as the different growth media are compared with each other (no control media).

**Figure 2.**
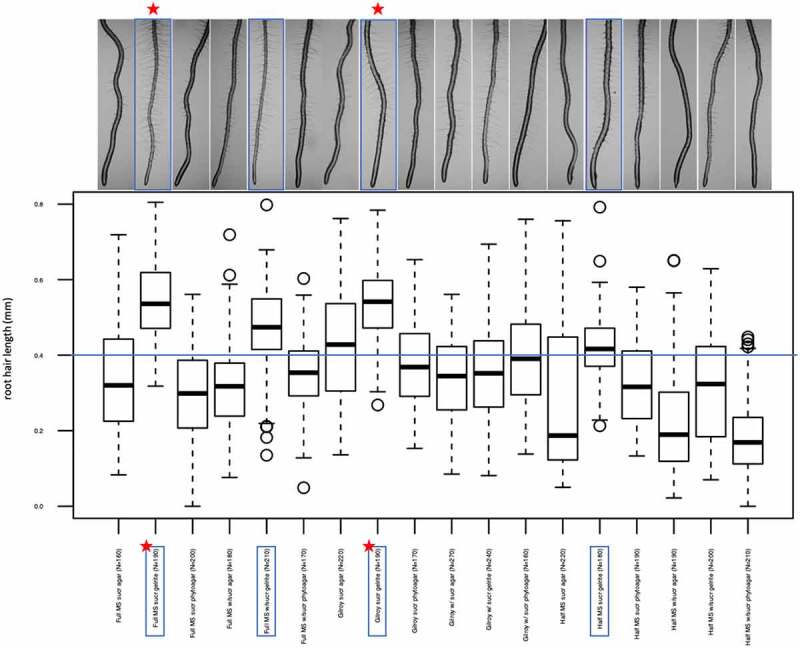
Boxplots representing root hair length of seedlings grown on one of the 18 different growth media. A representative image of the root and its root hairs on the respective medium is given above the graph. Those framed give the best results in terms of root hair length and density. Those with a red star give the best wild-type phenotype.

**Figure 3.**
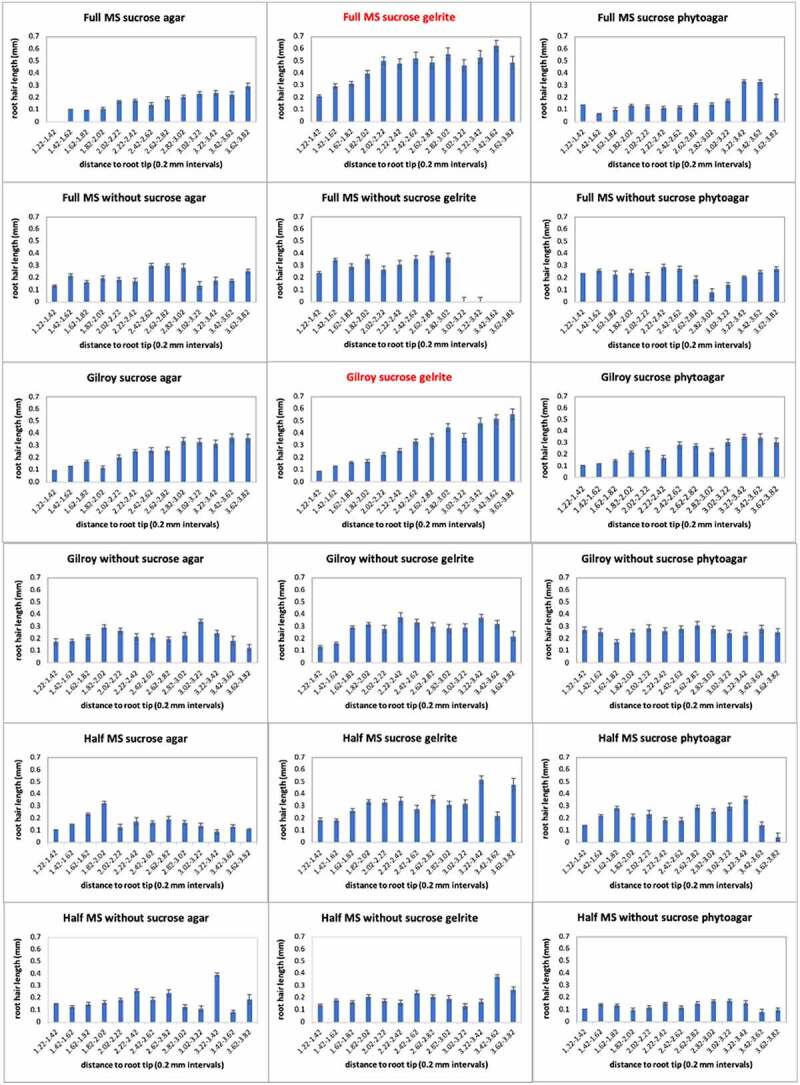
Graphical presentation of the root hair profile of wild-type on one of the 18 different growth media. Those in red give the best root hair profile according to the length of the root hairs (> 0.4 mm) and distance to the root tip (the further away from the tip, the longer the root hairs) .

As can be seen in the boxplot (Figure 2), there is a noticeable difference in root hair length on plants grown on the different growth media. Wild-type plants are best used as a control when their root hair length is longer than 0.4 mm and when they have a nice root hair profile (Figure 3). In other cases, it is not appropriate to use wild-type as a control to compare mutant lines, as this could give a distorted picture. The best wild-type profile can be obtained on full MS sucrose gelrite/full MS without sucrose gelrite/Gilroy sucrose gelrite and half MS sucrose gelrite (Figures 2 and 3). According to the boxplot, Gilroy media with sucrose and agar would also be a possible candidate because the root hairs are longer than 0.4 mm. However, the root hairs are not growing dense, just a few trichoblasts grow root hairs (as can be seen on the image provided above). The longest root hairs can be obtained when the seeds are grown on full MS sucrose gelrite or Gilroy sucrose gelrite. Taking all these data together, it can be said that both the addition of sucrose and the choice of the gelling agent are crucial for proper root hair growth. As has been shown by other researchers, sucrose has a positive effect on the length and number of root hairs, which can also be observed in this experiment. Besides sucrose, the gelling agent seems to have an impact. Root hairs will grow worse on media with agar or phytoagar as the gel is much more solid and loses more water. This excess water on the plates causes the roots to grow poorly without absorbing all the nutrients. Gelrite does not result in a very stiff gel, making it easier for the root hairs to grow longer. Importantly, the stiffness of course depends on the concentration of the gelling agent. In this experiment, however, the concentration of gelrite, agar and phytoagar was kept the same, i.e. 0.8% w/v.

## Difference in composition between growth media

As pointed out, there are several media available for growing plants *in vitro*.^6^ However, Murashige and Skoog (MS) medium is the most commonly used. It originates from White’s medium, but contains a higher concentration of all chemical compounds.^7^ As a consequence of these high concentrations of minerals, MS medium might be too salty for certain plant species as it is a very rich and saline medium. Therefore, powder of MS medium is available in which the concentration of macroelements is lowered with 50–75%, but the microelements are kept at the same level as described by 7. However, in this experiment the standard powder of MS including vitamins is used in which the concentration of macro-and microelements is remained the same as in the original MS medium.^7^ Since plants require the vitamin thiamine, the original MS vitamins can be replaced by the vitamins of Linsmaier and Gamborg B5 medium, as these contain a higher concentration of thiamine.^8,9^ Next to the popular MS medium, Gilroy medium is often used.^10^ This medium is a modified version of that of 11, and compared to MS medium, it contains more thiamine, but less of the microelements boric acid, zinc sulfate, manganese sulfate, and ethylenediaminetetraacetic acid. In addition, the concentration of the macroelements nitrate and ammonium is also considerably lower. In Table 1, the composition of the three most commonly used media is listed, allowing easier comparison.

**Table 1.** Summary of different media compositions. Colored boxes indicate a significant difference in the concentration of the relevant nutrient between the different media.

	Gilroy		Half MS		Full MS
Macroelements [mM]					
KNO_3_	3.00		9.40		18.79
Ca(NO_3_)_2_	2.00		– – – – – – – – –		– – – – – – – – –
MgSO_4_	0.50		0.75		1.50
NH_4_H_2_PO_4_	1.00		– – – – – – – – –		– – – – – – – – –
NH_4_NO_3_	– – – – – – – – –		10.31		20.61
KH_2_PO_4_	– – – – – – – – –		0.63		1.25
CaCl_2_	– – – – – – – – –		1.50		2.99
Total macroelements [mM]					
NO_3_^−^	7.00		19.70		39.40
NH_4_^+^	1.00		10.31		20.61
PO_4_^3-^	1.00		0.63		1.25
SO_4_^2-^	0.50		0.75		1.50
Ca^2+^	2.00		1.50		2.99
Mg^2+^	0.50		0.75		1.50
Microelements [μM]					
H_3_BO_3_	17.50		50.14		100.27
CuSO_4_.5H_2_O	0.25		0.05		0.10
ZnSO_4._7H_2_O	1.00		14.96		29.91
MnSO_4_.H_2_O	1.00		50.00		100.00
FeNaEDTA	25.00		50.00		100.00
KCl	25.00		– – – – – – – – –		– – – – – – – – –
KI	– – – – – – – – –		2.50		5.00
Na_2_MoO_4._2H_2_O	– – – – – – – – –		0.52		1.03
(NH_4_)_6_MoO_24_.4H_2_O	0.25		– – – – – – – – –		– – – – – – – – –
CoCl_2_.6H_2_O	– – – – – – – – –		0.06		0.11
Na_2_-EDTA	25		– – – – – – – – –		– – – – – – – – –
Vitamins [μM]					
Thiamine	3.00		0.15		0.30
Pyridoxine-HCl	2.43		1.22		2.43
Nicotinic acid	4.06		2.03		4.06
Glycine	– – – – – – – – –		13.32		26.64
Myo-inositol	554.94		277.47		554.94
Extra compounds					
MES [mM]	2.56	2.56		2.56	
Sucrose [mM]	29.21	29.21		29.21	
Gelling agent [w/v]	0.8	0.8		0.8	

## Multiple factors have an influence on the growth process

When studying root hairs, it is important to keep in mind that research groups are using different media to grow their *Arabidopsis* seeds. Therefore, it might be difficult to directly compare results and draw conclusions from the phenotypes of wild-type and mutant lines. There is a range of articles demonstrating the effect of certain factors on root hair growth, which is obviously linked to their absorptive function in the soil. In this paragraph, the effect of nutrients, sucrose, MES buffer, and the gelling agent will be discussed.

### Effect of available nutrients on root hair growth

MS medium contains 50–60 mM nitrogen. It is known that higher levels of nitrate (NO_3_^−^) lead to slower primary root growth, increased root branching, and an increase in root hair density, which, in turn, results in accumulation of nitrate in root hairs.^12–15^ However, if the nitrate concentration is too high, this is considered a disadvantage. The basipetal auxin fluxes are then limited, meaning inhibition of the flux of auxin to the root, creating a lower auxin concentration in the root and in addition a negative regulation of lateral root elongation.^12–16^ Ammonium (NH_4_^+^) is also found to be positive for root hair branching, yet it inhibits root growth and root hair elongation.^12–14^

As far as phosphate (P) is concerned, the opposite has been established. A lower concentration of phosphate results in a local up-regulation of auxin biosynthesis and ARF7/ARF19-induced *PHR1* expression, which results in a shorter root with more lateral roots and longer and denser root hairs.^14,17–19^ A similar effect as low P has been noticed in sulfur (S)-deficiency conditions.^20^ Under low sulfur supply the expression of SULTR;1 and SULTR1;2 is increased to stimulate sulfate uptake. As a consequence, the primary root length is shorter and more root hairs are formed to preserve root biomass. 21,have investigated the importance of extracellular calcium (Ca) during root hair growth. They have shown that maximal root hair length is achieved at a calcium concentration of 0.3–3.0 mM. A calcium influx at the tip of growing root hair cells has been demonstrated, and this results in a tip-focused calcium gradient. They have conclude that extracellular calcium is necessary to maintain polarized growth and to obtain normal root hair elongation.^21^ Regarding the response of root hairs to magnesium [Mg), 22,have found that their elongation is enhanced under low Mg conditions. On the contrary, their development is decreased progressively under high Mg supply. In this condition, the tip-focused reactive oxygen species and cytosolic calcium concentrations during elongation are lowered since new root hairs are formed due to the increased Mg concentration.

Boron (B] is an essential micronutrient for plants and it is taken up in the form of boric acid. Low boron supply leads to reduced primary root growth and more and longer root hairs, but a high concentration is toxic for the plant.^23^ Zinc (Zn) has a similar positive effect on root hair development as low boron supply. A high concentration of zinc causes branched and abnormally shaped root hairs, as it is toxic to the plant at high quantities.^24^ Regarding manganese (Mn), it seems that Mn-deficiency changes the arrangement and features of root epidermal cells. An increase in the frequency of root hairs can be noticed, as some additional and ectopic root hairs are formed from atrichoblasts. Apart from being denser, the root hairs are also longer under Mn-deficiency.^20,25^ Under low iron (Fe) conditions, root hair branching is stimulated rather than forming ectopic root hairs to increase their absorptive surface.^26,27^ Thus, it is important to keep in mind that root hair growth seems to be stimulated by the deficiency of phosphorus, boron, zinc, manganese, magnesium, sulfur and iron, whereas an excess of nitrate might have a negative effect.

With regard to vitamins, thiamine is the only one that shows a big difference in concentration between the two media. In Gilroy medium, the concentration of this vitamin is ten times higher compared to MS medium. Thiamine or vitamin B_1_ is involved in the processes underlying plant adaptations to stress (abiotic and biotic), but mainly oxidative stress.^28,29^ This might also apply for auxin as it seems to be involved in plant defense against stress. 30,have suggested that auxin might be the connecting link between regulating the level of ROS and directing its role in signaling or oxidative damage under stress.

### Effect of sucrose and MES

When it comes to sugar, several studies have shown that the addition of sucrose/glucose to the plant growth medium increases root length and the number of lateral roots, and alters root hair elongation.^31,32^ Moreover, sugar enhances indole-3 acetic acid (IAA) biosynthesis, a natural auxin in plants that among many other trophic responses, modulates the gravitropic response of the primary root.^32,33^,studied the influence of direct light illumination and sucrose supplementation on root length and root hair development/elongation. They have confirmed that dark-grown roots have a longer root compared to light-grown roots because of reduced stress responses in the root tip.^34^ Under direct illumination the total root length is inhibited but can be boosted by sucrose supplementation. This indicates that sucrose and light show an antagonistic effect when it comes to promoting root length. In that study, it was clear that sucrose supplementation triggered an increase in root hair length of light-grown roots. Taking together, continuous illumination and exogenous sugar act antagonistically on root length, but seem to have an additive effect on root hair outgrowth^33^

Besides sucrose, the buffer 2-[N-morpholino) ethanesulfonic acid, known as MES, is also added with a concentration of 2.56 mM. 35,have demonstrated that 51.2 mM of MES inhibits root growth, and decreases the number and length of root hairs, while 5.12 mM of MES has a stimulating effect. Furthermore, MES interferes with ROS homeostasis at 51.2 mM of MES as the generation of superoxides in the root apex disappears. This disturbance will affect normal root morphogenesis, given that ROS plays a crucial role in root growth (36]. An important objection is that 36,have not taken into account the effect of potassium (K) ions since they adjusted the pH after MES addition (requires up to 10 mM extra potassium). When plants are grown on potassium-deficient media, the production of ROS and ethylene is stimulated and the expression of the genes involved in ethylene biosynthesis and signaling is increased.^37,38^,have demonstrated that primary root growth is inhibited and root hairs grow longer on K+-deficient medium compared to K+-sufficient medium. It is known that root growth inhibition is a feature of ethylene’s triple response and ethylene also stimulates root hair elongation.,^14^

### Effect of gelling agent

To make each growth medium solid, a reagent, known as a gelling agent, is added to the liquid media. It acts as a ‘rock’, meaning that seedlings will encounter the hard surface and as a result will grow downward along the gravity vector when the plates are placed in a vertical position. The choice of gelling agent also has a profound influence on seedling growth. One would expect that gelling agents behave like an inert constituent of the plant’s growth medium, but the opposite is proven. Several studies have shown that plants grown on identical media, in which only the gelling agent is different, exhibit growth variations.^39^ All this is related to the differences in chemical and physical characteristics, such as gel strength, nutrient diffusion rate and organic and elemental impurities. In nutrient-rich media, the presence of elemental contaminants does not really affect growth. However, under nutrient-deficient conditions, these contaminants might have a significant effect on growth of Arabidopsis seedlings.^39^ There may in fact be a crosstalk between the available micro- and macroelements, which has implications for seedling development.^40,41^ It has been said that gelling agents can change the ion composition of the medium.^39^ Agar, which is obtained from the red alga *Rhodophyceae*, gives a very solid and stable gel. It has a gel strength of >1100 g/cm^2^. Gelrite is produced by microbial fermentation of the bacteria *Sphingomonas elodea* and results in a rigid (400–700 g/cm^2^), almost transparent gel, which facilitates visualization of root hairs. Moreover, it contains no contaminating products such as phenolic compounds that can be toxic for certain organisms. Phytoagar, another type of gelling agent, is free of the extra salts of other agars and has a high gel strength [950–1050 g/cm^2^).

### Effect may vary between ecotypes

**Figure 4.**
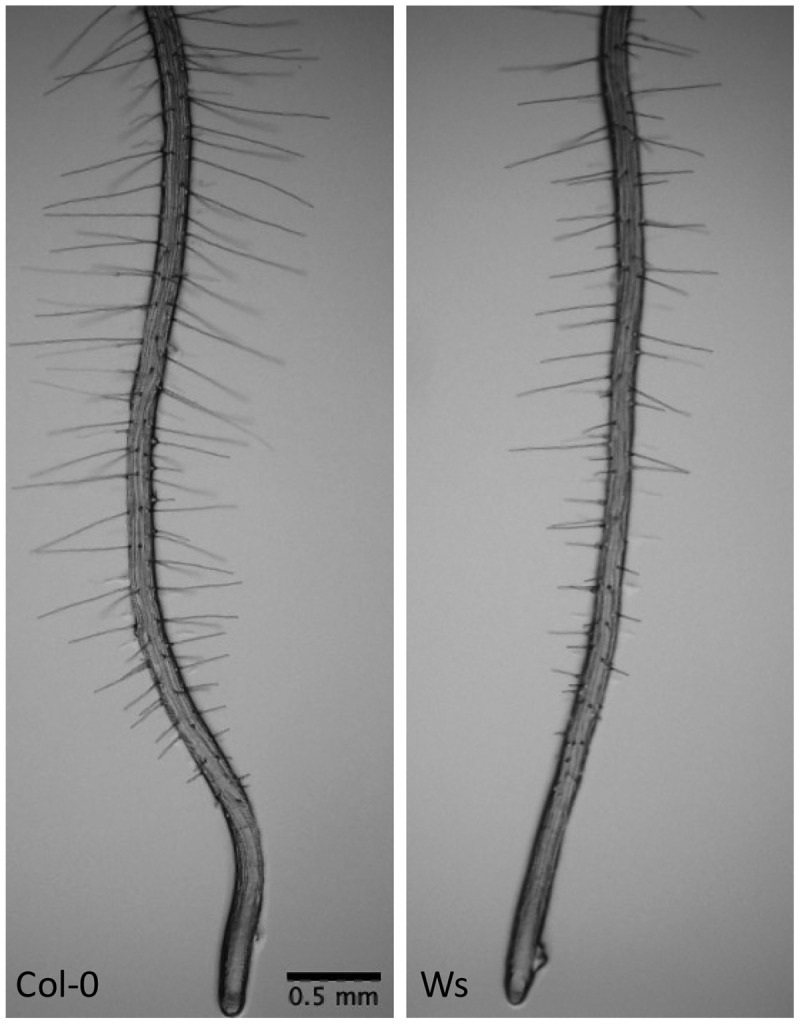
Difference in root hair phenotype between Columbia-0 (Col-0) and Wassilewskija (Ws) seeds that were grown in a growth chamber for 6 days at 21°C with 16/8 h light/dark photoperiod. Average difference in root hair length of 25%.

It is important to mention that in this experiment only the Columbia-0 wild-type is used, whereas many other ecotypes exist. 42,have shown that seedlings from a variety of *Arabidopsis thaliana* accessions exhibit a natural variation in root growth behavior. Regarding root hair length, an experiment has been performed in our lab in which Columbia-0 (Col-0] and Wassilewskija (Ws) seeds were grown on Gilroy media with sucrose and gelrite. As shown in Figure 4, a noticeable difference in root hair length can be observed between both accessions. Ws-root hairs are markedly shorter than Col-0 (up to 25% of Col-0 root hair length).

As such, it can be assumed that seedlings of various backgrounds will grow (slightly) different on the different growing media.

## Conclusion

As the data shows, the use of different growth media largely influences the development of Arabidopsis roots and especially root hairs. It would therefore, as a root (hair) community, be more efficient to use a consensus growth medium that eases the comparison of root and root hair phenotypes. This experiment would suggest to grow wild-type and mutant lines on either full MS with sucrose and gelrite or on Gilroy medium with sucrose and gelrite.
